# In Vivo Isolation and Characterization of Stem Cells with Diverse Phenotypes Using Growth Factor Impregnated Biomatrices

**DOI:** 10.1371/journal.pone.0001910

**Published:** 2008-04-02

**Authors:** Annalisa Grimaldi, Cristiano Bianchi, Gabriella Greco, Gianluca Tettamanti, Douglas M. Noonan, Roberto Valvassori, Magda de Eguileor

**Affiliations:** 1 Department of Structural and Functional Biology, University of Insubria, Varese, Italy; 2 Department of Clinical and Biological Sciences, University of Insubria, Varese, Italy; 3 IRCCS Multimedica, Milan, Italy; Ordway Research Institute, United States of America

## Abstract

**Background:**

The stimulation to differentiate into specific cell types for somatic stem cells is largely due to a series of internal and external signals coming from the microenvironment that surrounds the stem cell. Even though intensive research has been made, the basic mechanisms of plasticity and/or the molecules regulating stem cells proliferation and differentiation are not completely determined. Potential answers concerning the problems could be derived from the studies of stem cells in culture.

**Methodology/Principle Findings:**

We combine a new procedure (using the matrigel biopolymer supplemented with a selected cytokine/growth factor) with classic techniques such as light, confocal and electron microscopy, immunohistochemistry and cell culture, to perform an analysis on stem cells involved in the leech (*Hirudo medicinalis)* repair tissues. The leech has a relative anatomical simplicity and is a reliable model for studying a variety of basic events, such as tissue repair, which has a striking similarity with vertebrate responses. Our data demonstrate that the injection of an appropriate combination of the matrigel biopolymer supplemented with a selected cytokine/growth factor in the leech *Hirudo medicinalis* is a remarkably effective tool for isolating a specific cell population in vivo. A comparative analysis of biopolymer *in vivo* sorted stem cells indicates that VEGF recruited cells of a hematopoietic/endothelial phenotype whereas MCP-1/CCL2 isolated cells that were of an early myeloid lineage.

**Conclusion:**

Our paper describes, for the first time, a method allowing not only the isolation of a specific cell population in relation to the cytokine utilized but also the possibility to culture a precise cell type whose isolation is otherwise quite difficult. This approach could be broadly applied to isolate stem cells of diverse origins based on the recruitment stimuli employed.

## Introduction

All adult multicellular organisms with either simple or complex body plans harbor somatic stem cells. These are undifferentiated cells found throughout the body that are able to renew themselves through mitotic processes, and have the capacity to generate daughter cells able to differentiate into a variety of specialized cells [1], [2]. In mammals, adult stem cells occur in numerous organs and tissues and are characterized by a remarkable level of plasticity (i.e. stem cells from one tissue can give rise to cells of different tissue, for example haematopoietic stem cells can form not only all types of blood cells, but also can give rise to muscle cells or to neurons under specific conditions) [3], [4]. The stimulation to differentiate into a specific cell type for somatic stem cells appears to largely be a series of internal and external signals, which can include chemical and molecular signals such as cytokine and growth factor secretion within a predetermined microenvironment that surrounds the stem cell and provides determinant clues [5]

The plasticity of somatic stem cells has stimulated great interest due to their numerous potential applications, in addition to basic and in clinical research, as a substitutive or regenerative therapy for numerous human diseases or injuries [6], [7]. A subject of intensive research, many problems remain in the adult stem cell field. Among these, one important point is that the somatic stem cells that have been identified in most organs and tissues including skin, liver, brain, bone marrow, blood vessels, muscles, are always present in poor numbers. In addition, the possible sources of somatic stem cells, their percentage in various tissues and often their origins are as yet not well defined and the basic mechanisms of plasticity and/or the molecules regulating the proliferation and the differentiation are not completely determined. Potential answers concerning these problems could be derived from studies of somatic stem cells in culture. Clearly cell culture would be an invaluable and potent method for studying somatic stem cells from a morphological, immunocytochemical, biochemical and molecular point of view, although many technical hurdles must be overcome: in fact it is difficult to isolate somatic stem cells from the surrounding tissues, and to find markers to characterize them.

Here we have focused on an alternative animal model, the leech (*Hirudo medicinalis*), which has the important advantages of being an economic invertebrate suitable for experimental manipulation, easily manipulated and without significant emotional or regulatory restrictions. The leech has a relative anatomical simplicity (the body consists of a muscular-cutaneous sac which contains several organs embedded in a loose connective tissue) and is a reliable model for studying a variety of basic events, such as tissue repair, which has a striking similarity to those of vertebrate responses [8], [9], [10], [11], [12] even if the major draw-back to this model to date has been the difficulty in isolating cells for culture and maintenance of sterility.

Although the wound healing process in leeches is a dynamic continuum, however, it can be classified into three principal phases consisting of a complex series of overlapping events. The first event is the inflammatory phase, followed by a proliferative phase with the formation of a granulation tissue, and by a final maturation phase. In particular, epithelium formation, angiogenesis, and collagen deposition are the principal steps in the anabolic phase of wound healing. During these phases, a number of secreted enzymes and cytokines modulate phenotypic changes, mitogenesis and migration of different types of cells. Several studies have previously demonstrated the importance of a number of cytokines, including VEGF, EGF, bFGF, GM-CSF and TGF-β, during angiogenesis and fibroplasia in this animal model [13], [14]; Grimaldi et al. unpublished data). Most of these events, often concomitant, are shared by vertebrates and invertebrates, leading [15] to suggest that the genetic pathways regulating tissue formation are highly conserved, and that the tissue repair cascade may be identical to the pathways that delineate mesodermal tissue formation and organization in invertebrates as well as vertebrates.

Despite the fact that vertebrate and invertebrate tissue repair and related phases such as angiogenesis, associated haematopoiesis, and cytokine implications are well known processes *in vivo*, *in vitro* studies on to role of stem cells in these processes are to date quite limited. This is mostly due to the difficulty in isolation of somatic stem cells from tissues in significant numbers.

Here we describe a method that allows isolation of a specific cell populations that also simplifies culture of cell types *in vitro* whose isolation and culture is otherwise quite difficult. This method is not only invaluable for studying and charactering the cells involved in wound healing responses but also could be a simple system for select specific cell populations *in vivo* to then culture them *in* vitro.

## Results

**Figure 1:**
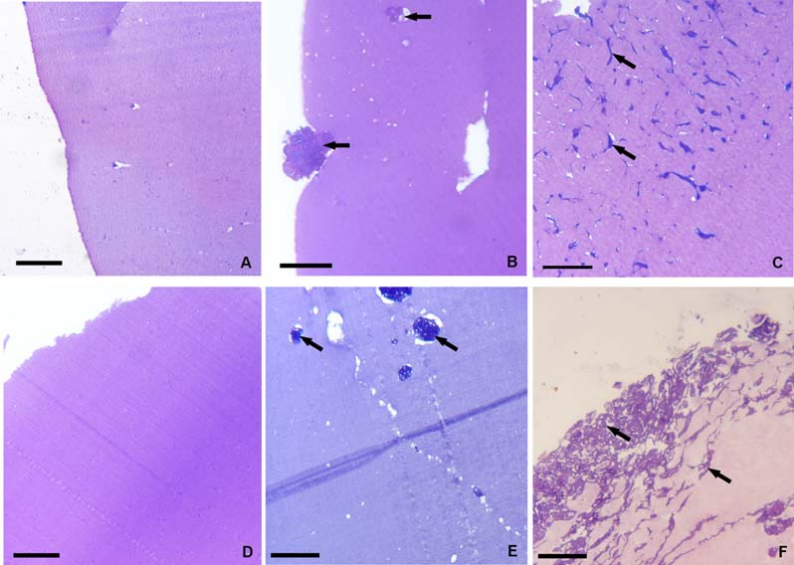
Histology of matrigel sponges *in vivo*. The cells infiltrating the gels without (A, D) and with the cytokines -VEGF (B, C) or MCP-1/CCL2 (E, F) after 48 h (A, B, E) and 1 week (D, C, F). The two cytokines selectively recruit different cellular types. Bars: A–F, 20 µm

The Matrigel sponges which had formed following inoculation were recovered and processed for standard histology after 48 h, and 1 week. On examination, few migrating cells were seen in control Matrigel specimens lacking growth factors, whereas Matrigel specimens containing either vascular endothelial growth factor (VEGF) or Monocyte chemoactractant protein-1 (MCP-1/CCL2) were “colonized” by an increased number of cells in relation to the time elapsed from the injection of the supplemented biomatrix (Fig.1 A–F).

### VEGF supplemented Matrigel

#### Light and electron microscopic examination

**Figure 2:**
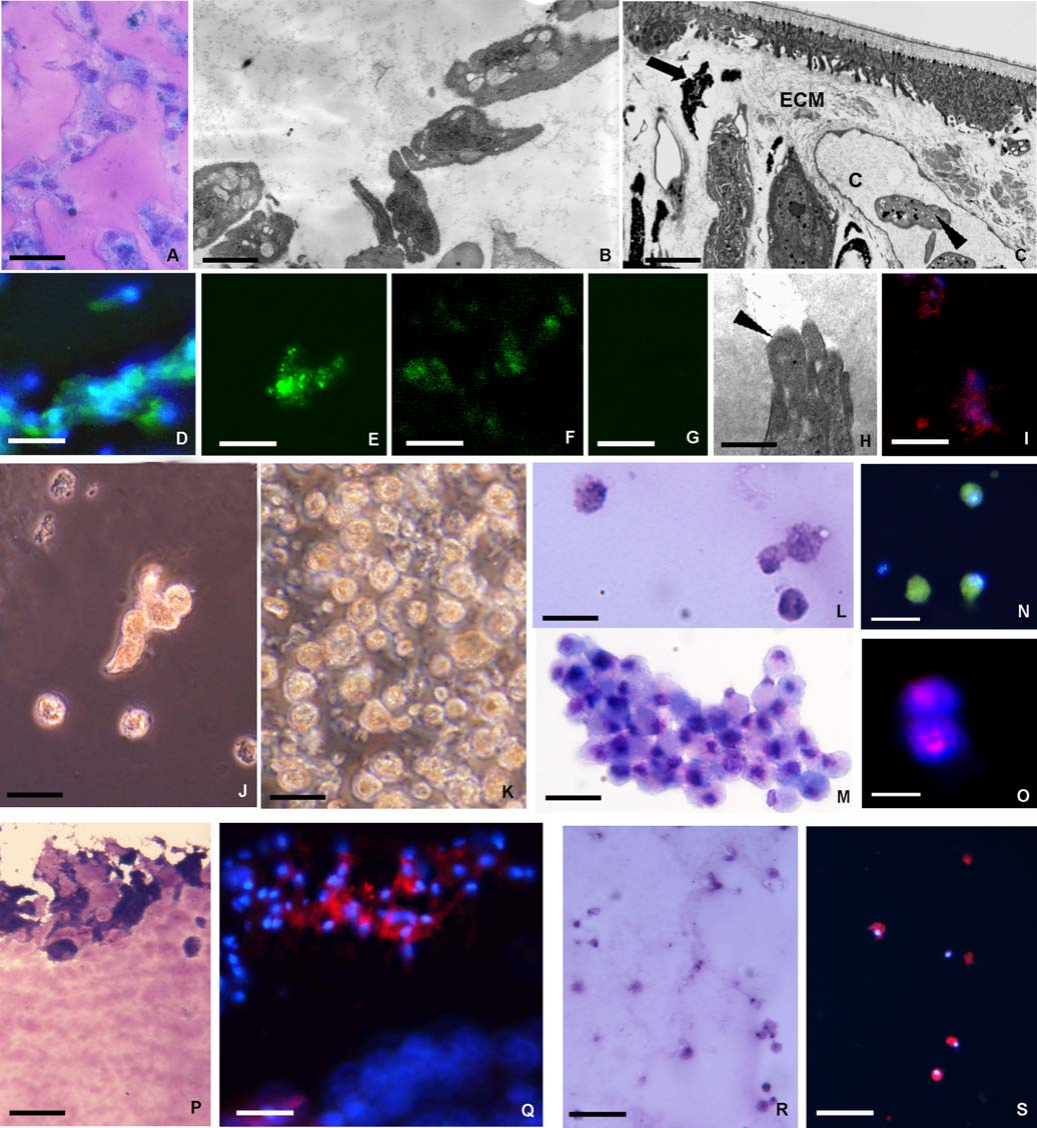
Analysis of the phenotype of cells recruited into the matrigel sponges by VEGF. VEGF induces the mobilization of cells from tissues surrounding the MG implant into the biopolymer that was recovered and processed for structural, ultrastructural immunocytochemical analysis or seeded for cell culture. After 1 week *in vivo* the matrigel plug contained hematopoietic precursor cells stained with Giemsa solution (A) that were ultrastructurally similar (B) to those localized in capillary (c) and/or in the extracellular matrix (ECM) in stimulated leeches (arrowheads in C). These cells were small in size with large nucleus and scarce cytoplasm (B). Immunocytochemical characterization of cells infiltrating the MG supplemented with VEGF (D–G). The cells expressed several markers typical of hematopoietic/endothelial cells: CD34 (D), CD117 (E), CD31 (F); (G: negative control). The cells showed a migratory phenotype with degradation of ECM that was associated with their cytoplasmic projections (H, arrowhead), were also cathepsin B positive (I). After 1 week *in vivo* the MG infiltrated with precursor cells was removed and used to seed cell cultures (J–O). Phase-contrast (J, K) and May Grunwald Giemsa staining (L, M) micrographs, obtained 3 days (J, L) and 1 week (K, M) post-seeding, shows the increasing cell numbers from the initially few and scattered (J, L) to growth in clusters (K, M). Immunofluorescence detects expression of CD34 (N), a marker of precursor cells, of the cells label with BrdU indicating that these cells have cycled through S-phase (O). After incubation with Dil-Ac-LDL, those cells that localized in the superficial area of MG implant were Giemsa positive (P) took up fluorochrome-conjugated Ac-LDL (Q). Identical results were found within the monolayer of these cultured cells that were Giemsa and DIL positive (R, S). Taken together, these results strongly indicate that the cells migrating into the MG in vivo growing out in vitro consisted essentially of precursor/endothelial like cells. Bars: A 20 µm; B 2 µm; C 4 µm; D-N, P, Q 10 µm; O 5 µm; R, S 50 µm.

Sections showed that small cells migrated from the periphery towards the matrix centre where they formed cell aggregates (Fig. 1 A–C; Fig. 2 A–B). These cells, similar to those described as haematopoietic precursor cells at the stage of granulation tissue formation during leech wound healing (Fig. 2 C) [8], were present in different percentages. The cells appeared morphologically similar at the two times considered (48 h and 1 week), while their numbers increased starting from 48 h up to one week from the biomatrix injection time (Fig. 1 A–C). The migrated cells were positive for May Grunwald Giemsa differential staining (Fig. 2 A) and appeared morphologically to be stem cells based on their similarity with stem cells *in vivo* (Fig. 2 C) and on the small dimensions characterized by large nuclei with a scarce cytoplasm occupied by organules and few small granules (Fig. 2 B).

#### Immunocytochemical characterization

Under the influence of VEGF the cells that infiltrated the matrigel exhibited a undifferentiated, highly migratory phenotype. Using a panel of antibodies, the cells were positive for markers expressed by hematopoietic stem cells including CD34 and CD117 (c-kit) as well as the endothelial marker CD31 (Fig. 2 D–G), suggesting an early hemangioblast phenotype. The cells were instead negative for a series of myeloid markers including CD14, CD68, CD61 and CD11c (Table 1). The cells were associated with, in proximity to their cytoplasmic projections, a degraded extracellular matrix (Fig. 2H) that corresponded to a strong cytoplasmic positivity for Cathepsin B (Fig. 2 I).

**Table 1:** Cells recruited by VEGF or by MCP-1 express different markers

	CD31	CD34	CD117	CD14	CD68	CD61	CD11c
MG+VEGF	+	+	+	-	-	-	-
MG+MCP1/CCL2	-	-	-	+	+	+	+

Cells infiltrating the matrigel (MG) supplemented with VEGF expressed only hematopoietic stem cells markers and are negative for myeloid lineage markers. On the contrary, cells that migrated into the matrigel supplemented with MCP-1/CCL2 expressed only myeloid lineage markers.

#### Cultured cells

Starting from 3 days (Fig. 2 J) up to 1 week (Fig. 2 K) after seeding the cells were present as either single cells or clusters (Fig. 2 J–M). These cells, positive for May Grunwald Giemsa differential staining (Fig. 2 L, M), exhibited both the morphological aspects described for the cells within the MG *in vivo* and the expression of the same set of specific surface markers of hematopoietic precursors such as CD34 (Fig. 2 N; Table 1). These cells were replicating as validated by BrdU incorporation (Fig. 2 O).

#### Vital dye analysis

After incubation with Dil-Ac-LDL, these cells took up the fluorochrome-conjugated probe (Fig. 2 P–S), indicating scavenger receptor expression typical of endothelial cells or macrophages. Cytoplasmic localization was evident in both *in vivo* experiments where the labeled migrated cells were visible at the edges of MG and within the VEGF supplemented biopolymer (Fig. 2P–Q), and in the cultured cells *in vitro* (Fig. 2 R, S).

### MCP-1/CCL2 supplemented Matrigel

#### Light and electron microscopic examination

**Figure 3:**
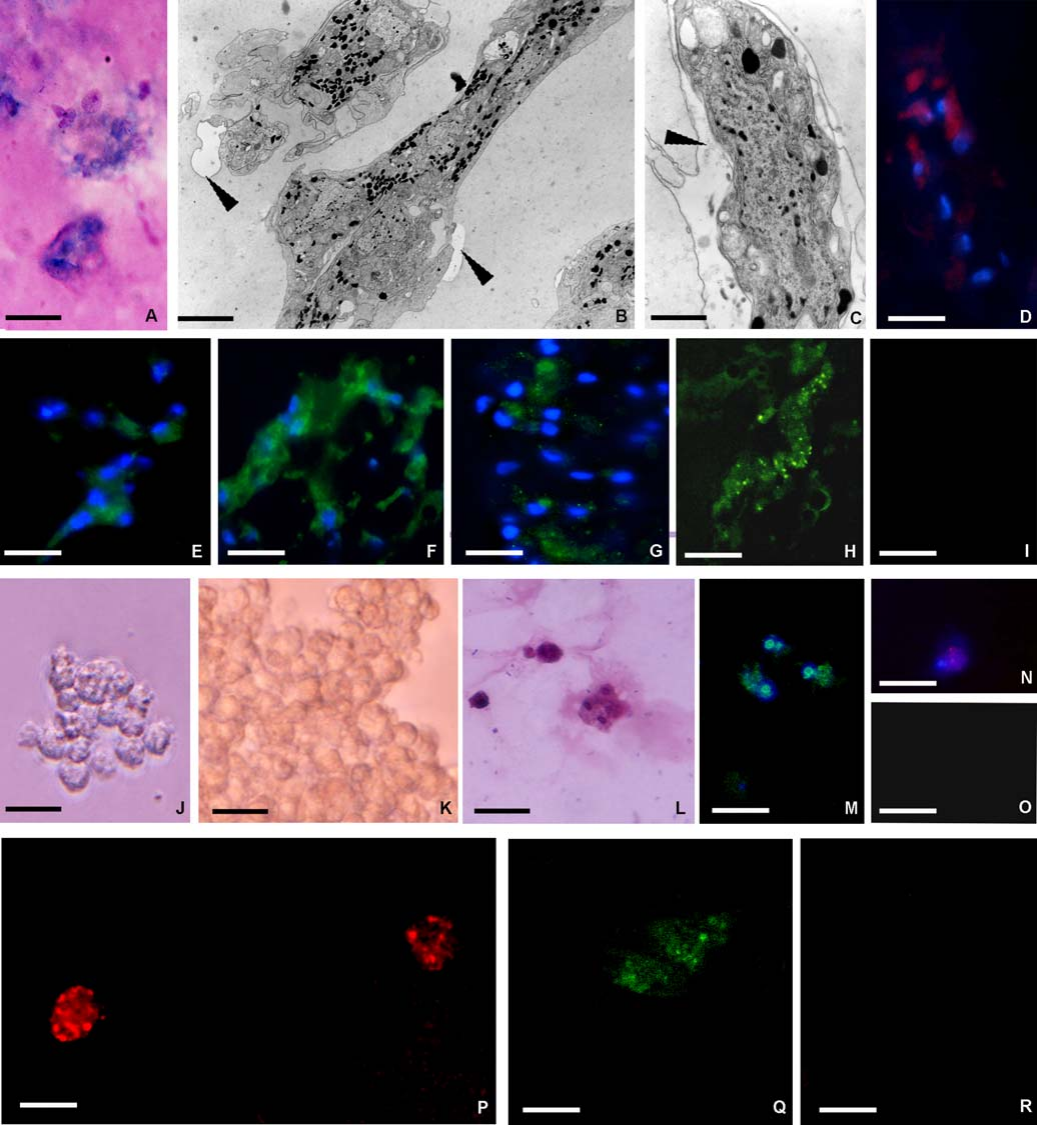
Analysis of the phenotype of cells recruited into the matrigel sponges by MCP-1/CCL2. MCP-1/CCL2 induces the mobilization of cells from tissues surrounding the MG implant into the biopolymer that was recovered and processed for structural, ultrastructural immunocytochemical analysis or seeded for cell culture (A–O). After 1 week *in vivo* the matrigel implant contained granular cells that stained with Giemsa solution (A) that were ultrastructurally similar (B) to those localized in the extracellular matrix (ECM) in stimulated leeches (Fig. 2C, arrowhead). These cells showed a cytoplasm filled with black granules (B, C). Again they were of a migratory phenotype with localized degradation of ECM (B, C arrowheads), and cathepsin B staining (D). Immunocytochemical characterization of cells infiltrating the MG supplemented with MCP-1/CCL2 (E–I). The cells expressed several markers typical of granulocytes: CD11c (E), CD14 (F), CD61 (G), CD68 (H); (I: negative control). After 1 week *in vivo* the MG was used to seed cell cultures (J–O). Phase-contrast images of granular cells obtained 3 days (J) and 1 week (K) post-seeding indicate increasing cell numbers (K). These Giemsa positive cells (L) also immuno-stained with a CD11c antibody (M), a marker of granulocytes, and with anti-BrdU (N) demonstrating that these cells attraversed S-phase in culture; O: negative control. After incubation with Dil-Ac-LDL, these cells also took up the fluorochrome-conjugated Ac-LDL (P). These results strongly indicate that the cells that migrated out of the matrigel *in vitro* consisted largely of cells maintaining a monocyte/macrophage function throughout *in vivo* and *in vitro* culture as indicated by expression of CD68 (Q, R). Bars: A, D, P–R 10 µm; B, C 1 µm; E–I 15 µm; J–O 20 µm

Light microscopy indicated that only one type of cell was present. These cells, which were morphologically different from those observed invading the matrigel in response to VEGF, exhibited the same appearance both at 48h and 1 week. Their number increased over time *in vivo* (Fig. 1 D–F). These large cells were elongated, and adopted a pronounced migratory and spreading phenotype (Fig. 3 A–C). This cellular phenotype appeared to invade into the surrounding extracellular matrix that showed clear signs of degradation (Fig. 3 B, C). These cells were characterized by cytoplasm filled with granules of diverse sizes (Fig. 3 B, C) similar to one of the cell types described in the granulation tissue during leech wound healing (Fig. 2 C).

#### Immunocytochemical characterization

By immunolocalization studies, MCP-1/CCL2 attracted cells with a highly migratory phenotype showing a strong positivity for Cathepsin B (Fig. 3 D) and expressing high levels of CD11c, CD14, CD61, and CD68, indicating a myeloid phenotype (Fig. 3 E–I; Table 1). Interestingly, they were also positive for endothelial marker CD31, but not for CD34 or CD117, indicating a pleiotropic, more mature phenotype than the cells recruited by VEGF (unpublished data).

#### Cultured cells

Starting from three days up to 1 week after seeding (Fig. 3 J–L), the cultured cells maintained the same morphology, appeared closely adherent among themselves and were positive for May Grunwald Giemsa differential staining (Fig. 3 L). The cells exhibited the expression of a similar set of specific markers, such as CD11c (Fig. 3 M; Table 1), as that described for those infiltrating into the MG *in vivo*. These cells were as also proliferating as indicated by BrdU incorporation (Fig. 3 N, O).

#### Vital dye analysis

After incubation with Dil-Ac-LDL, the cells attracted by MCP-1/CCL2 took up the fluorochrome-conjugated probe (Fig. 3 P) showing a spotted localization. This indicated that they maintained their monocyte/macrophage function throughout the *in vivo/in vitro* culture as also indicated by expression of CD68 (Fig. 3 Q, R).

## Discussion

Previous experimental studies have suggested that inclusion of specific angiogenic chemokines in matrigel injected *in vivo* using murine models is able to promote a suitable microenvironment for recruitment of specific cell infiltrates [4], [16], [17]. Here we show that injection of an appropriate combination of a matrigel biopolymer supplemented with a selected chemokine or growth factors in the leech *Hirudo medicinalis* is a remarkably effective tool for isolation specific cell populations *in vivo*. When the biomatrix was supplemented with Vascular endothelial growth factor (VEGF), we selected for cell populations that have the characteristics of hematopoietic stem cells. If we changed the factor incorporated into the polymer to Monocyte chemoactractant protein-1 (MCP-1/CCL2), we selected out a stem cell population with phenotypically quite different characteristics. While this method could likely be readily extended to murine of other animal models for isolation of specific cell types from surrounding tissues, we also note that this method allows ready sterile isolation of cells in conditions that are otherwise extremely difficult. Finally we propose that the obtained biopolymer infiltrated with the specific cell population can be utilized to “vector” cells from animal to tissue culture.

The factors used were chosen for initial studies because they play a major role in invertebrate/vertebrate wound healing and are known to stimulate stem cells in mammalian and invertebrate models [12], [18]. Matrigel with VEGF at early time points showed a high cell density due to the recruitment of hematopoietic precursor cells. These cells appeared morphologically to be stem cells and expressed CD34, CD117, and CD31, markers commonly identifying vertebrate hematopoietic, myeloid and leukocyte lineages.

In contrast, matrigel with MCP-1/CCL2 selectively sorted large, granule containing cells, with a pronounced migratory and spreading phenotype, that expressed high levels of CD11c, CD14, CD68, and CD61.

Both types of cells readily phagocytosed labeled acetylated LDL, an activity typical of cells from the endothelial and macrophage lineages, in agreement with the immuno-histochemistry indicating precursor hematopoietic/endothelial origin of the cells recruited by VEGF and a more myeloid/macrophage lineage of the cells recruited by MCP-1.

Matrigel specimens containing either cytokine were “colonized” by increasing numbers of cells in relation to the time elapsed after supplemented biomatrix injection. In both cases (MG supplemented with VEGF or MCP-1/CCL2), the biopolymer was populated by cells actively dividing as demonstrated by BrdU incorporation.

Unlike standard methods to localize stem cells in adult tissues, to extract and culture them, the present approach provides a method by which adult stem cells move from the tissues in which they reside to reach the injected matrigel supplemented with a specific cytokine. Afterwards, the biopolymer attains a high cell density due to recruitment of specific stem cell populations can be used as a vector to prepare cell cultures. After 48h *in vivo,* we were able to remove the matrigel biopolymer containing the migrated cells from the body of animals under sterile conditions and culture the implants *in vitro*. This allowed us to isolate specific cell populations *in vitro* with the same appearance and expressing the same specific markers (both superficial and cytoplasmic) as found *in vivo*, suggesting that the staminal cell nature of the cells infiltrating the matrigel can be preserved upon passage into culture.

To the best of our knowledge, this study describes, for the first time, a method allowing not only the isolation of a specific cell population in relation to the cytokine utilized but also the possibility to culture these precise cell types whose isolation has been very difficult to date. This method will not only be invaluable for studying and charactering cells involved in leech wound healing responses but it could be also an easy system to select specific cell populations for *in vitro* culture, expansion and differentiation analyses.

## Materials and Methods

### Animals and treatments

Leeches (*Hirudo medicinalis*, Annelida, Hirudinea, from Ricarimpex, Eysines, France) measuring 10×1.00 cm were kept in water at 19–20°C in aerated tanks. Animals were fed weekly with calf blood. Animals were randomly divided into separate experimental groups (see below) according to different protocols and treatments.

Before each experiment, leeches were anaesthetized with an 10% ethanol solution. Leeches were injected with liquid Matrigel (MG) supplemented or not with cytokines under dorsal tegument at the hind part of the animal, about 2/3 from the oral extremity (at about the 80^th^ superficial metamere).

Matrigel (MG), an extract of the murine Engelbreth-Holm-Swarm (EHS) tumor grown in C57/b16 mice, was produced as described by [19]. MG is a complex biomatrix rich in basement membrane components (laminin, collagen IV, nidogen, and perlecan) is a thermo sensitive material liquid at 4°C yet polymerizes when warmed to room temperature. This biomatrix was supplemented for each experiment with different types of growth factor/cytokines selected among those that play a major role in invertebrate/vertebrate wound healing: Vascular Endothelial Growth Factor (VEGF), among the most potent angiogenic and haematopoietic factors, and Monocyte chemoactractant protein-1 (MCP-1/CCL2) a chemokine with a pivotal role in the recruitment of monocytes and macrophages.

#### In vivo Matrigel assays

88 animals were subdivided into three groups as follows: Group (1): control animals, 8 leeches injected with MG only. Group (2): 40 leeches were injected with 300 µl of matrigel added with 50 ng of vascular endothelial growth factor (VEGF) (Pepro Tech, London). Group (3): 40 leeches were injected with 300 µl matrigel with 50 ng of MCP-1/CCL2 (Pepro Tech). Each anesthetized leech belonging to the three groups received one injection of MG. Anesthetized leeches were dissected and polymerized matrigel pellets were removed at the various time points as indicated. In particular, the injected 40 animals of Groups 2 and 3 were further subdivided into two subgroups; 4 control animals and 20 animals belonging to subgroups 2a and 3a from which the matrigel pellets were recovered after 48 h (eight animals), or 1 week (eight animals), from injection, were histologically, ultrastructurally and immunocytochemically examined. In another series of 4 control animals and 20 leeches belonging to subgroups 2b and 3b were anesthetized and dissected for cell culture.

#### In vitro Matrigel assays

After 1 week *in vivo* (corresponding to a suitable cell concentration for seeding) Matrigel implants were harvested and cultured. Each matrigel pellet was minced in small pieces using sterilized razor blades and plated in wells of 60 mm in diameter in DMEM medium (Celbio, Milan, Italy) modified by dilution (1:4) to reach iso-osmolality and supplemented with 1% glutamine and 10% fetal bovine serum. Cells were maintained at 20°C and histologically and immunocytochemically examined 3 days and 1 week after seeding.

#### Optical and Electron microscopy

Samples were fixed for 2 h in 0.1 M cacodylate buffer pH 7.2, containing 2% glutaraldehyde. Specimens were then washed in the same buffer and postfixed for 2 h with 1% osmic acid in cacodylate buffer, pH 7.2. After standard serial ethanol dehydration, specimens were embedded in an Epon-Araldite 812 mixture. Sections were obtained with a Reichert Ultracut S ultratome (Leica, Wien, Austria). Semithin sections were stained by conventional methods (crystal violet and basic fuchsin) according to Moore et al. (1960), and by differential staining (May Grunwald Giemsa) to permit identification of hematopoietic cells, and subsequently observed under a light microscope (Olympus, Tokyo, Japan). Thin sections were stained by uranyl acetate and lead citrate and observed with a Jeol 1010 EX electron microscope (Jeol, Tokyo, Japan).

#### Indirect immunofluorescence staining

Matrigel implants were embedded in Polyfreeze tissue freezing medium (Polysciences, Eppelheim, Germany) and immediately frozen in liquid nitrogen. Cryosections (7 µm) were obtained with a Leica CM 1850 cryotome and slides were immediately used or stored at −20°C. Samples washed with PBS were incubated with primary antibodies (diluted 1:20) to either anti-CD11c, CD14, CD33, CD34, CD61, CD68, CD117 (Santa Cruz Biotechnology, CA, USA) and anti Cathepsin B (Sigma, St. Louis, MO) for 1 h at room temperature. The washed specimens were incubated for 1 h at room temperature with the appropriate secondary antibody (Jackson, Immuno Research Laboratories, West Grove, PA, U.S.A.) Cy3 or FITC (fluorescein isothiocyanate) conjugated (dilution 1∶100). Nuclei were stained by incubating for 15 min with 4′,6-Diamidino-2-Phenylindole (DAPI, 0.1 μg/ml in PBS). The PBS buffer used for the washing steps and antibody dilutions contained 2% bovine serum albumin (BSA). The slides were mounted in Citifluor (Citifluor Ltd, London, UK) with coverslips and examined with fluorescence microscope Olympus BH2 (Olympus). The staining was visualized using excitation/emission filters 550/580 nm for Cy3, 490/525 nm for FITC and 340/488 nm for DAPI. Data were recorded with a DS-5M-L1 digital camera system (Nikon, Tokyo, Japan). Images were combined with Adobe Photoshop (Adobe Systems, Inc.).

In control samples, antibodies were omitted and sections were treated with BSA-containing PBS.

#### Vital dye injection

We used injection of the vital dye Acetylated Low Density Lipoprotein (Dil-Ac-LDL) (Biomedical Technologies Inc., MA, USA) a functional marker for endothelial cells and macrophages to functionally characterize the cells migrating into the biopolymer. According to the manufacturer's suggestions and previous studies [20] in *in vivo* experiments, injection of 10 µl of Dil-Ac-LDL (10 µg/ml) in PBS buffer (0.01 M) was made at the level of the 80^th^ superficial metamere where the MG was subsequently inoculated. After 1 week the MG implant was removed from the animal as described above and quick frozen. Cryosections (7 µm) were mounted in Citifluor (Citifluor Ltd) and mounted slides were viewed on fluorescence microscope Olympus BH2 through a rhodamine filter set to visualize the Dil (excitation/emission filters 550/580 nm). Images were acquired with a DS-5M-L1 digital camera system Nikon.

For *in vitro* experiments, cells were cultured in DMEM containing 10 µg/ml Dil-Ac-LDL according to the manufacturer's suggestions and [21]). After 4 h at room temperature (RT), cells were washed several times with probe-free media and directly observed using an inverted-fluorescence microscope (Olympus). The reaction was visualized using standard rhodamine excitation/emission filters (550/580 nm). Data were recorded with a DS-5M-L1 digital camera system (Nikon). Experiments were performed in triplicate in both *in vivo* and *in vitro* analyses.

#### Proliferation assay

We used an immunocytochemical system for monitoring cell proliferation using monoclonal anti-5-bromo-2′-deoxyuridine (BrdU) to detect BrdU incorporation into cellular DNA. Cells were treated according to manufacturer's protocol (Amersham-Pharmacia, Buckinghamshire, UK) and examined with Olympus BH2 microscope (Olympus). Controls were performed by omitting the primary antibody.
